# Author Correction: Multi-omics analysis in human retina uncovers ultraconserved cis-regulatory elements at rare eye disease loci

**DOI:** 10.1038/s41467-024-48497-6

**Published:** 2024-05-10

**Authors:** Victor Lopez Soriano, Alfredo Dueñas Rey, Rajarshi Mukherjee, Genomics England Research Consortium, Chris F. Inglehearn, Frauke Coppieters, Miriam Bauwens, Andy Willaert, Elfride De Baere

**Affiliations:** 1 Department of Biomolecular Medicine, Ghent University, Ghent, Belgium; 2 Center for Medical Genetics, Ghent University Hospital, Ghent, Belgium; 3 Department of Ophthalmology, St James’s University Hospital, Leeds, UK; 4 Department of Pharmaceutics, Ghent University, Ghent, Belgium; 5 Division of Molecular Medicine, Leeds Institute of Medical Research, University of Leeds, Leeds, UK

**Keywords:** Gene regulation, Epigenomics, Data mining

Correction to: *Nature Communications* 10.1038/s41467-024-45381-1, published online 21 February 2024

The original version of this Article contained an error in the Acknowledgements, which incorrectly omitted the following: ‘This publication is part of the Human Cell Atlas - www.humancellatlas.org/publications (HCA-51)’. This has been corrected in both the PDF and HTML versions of the Article.

