# Supplementary material for: Multi-omics analysis in human retina uncovers ultraconserved cis-regulatory elements at rare eye disease loci
Source: Nat Commun. 2024 Feb 21;15:1600. doi: 10.1038/s41467-024-45381-1 (PMC10881467; doi:10.1038/s41467-024-45381-1)
Supplement: Supplementary file 1 — Supplementary Information file [file 41467_2024_45381_MOESM1_ESM.pdf]

Supplementary Material

Multi-omics analysis in human retina uncovers ultraconserved *cis*-regulatory elements at rare eye disease loci

Victor Lopez Soriano[*1,2], Alfredo Dueñas Rey[*1,2], Rajarshi Mukherjee[3], Genomics England Research Consortium[4], Frauke Coppieters[1,2,5], Miriam Bauwens[1,2], Andy Willaert[1,2], Elfride De Baere[1,2]

1- Department of Biomolecular Medicine, Ghent University, Ghent, Belgium.
2- Center for Medical Genetics, Ghent University Hospital, Ghent, Belgium.
3- Department of Ophthalmology, St James's University Hospital, Leeds, UK.
4- Genomics England Research Consortium, UK. Rajarshi Mukherjee, Department of Ophthalmology, St James's University Hospital, Leeds, UK; Chris F. Inglehearn, Division of Molecular Medicine, Leeds Institute of Medical Research, University of Leeds, Leeds, UK.
5- Department of Pharmaceutics, Ghent University, Ghent, Belgium.

*equal contribution

Corresponding author: Elfride De Baere (elfride.debaere@ugent.be)

Supplementary Figures

a

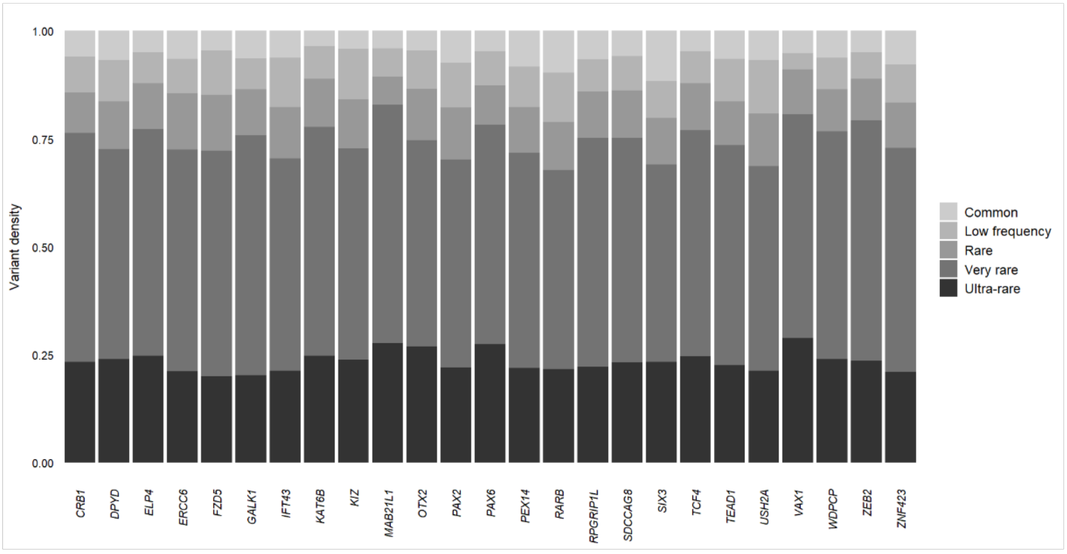

b

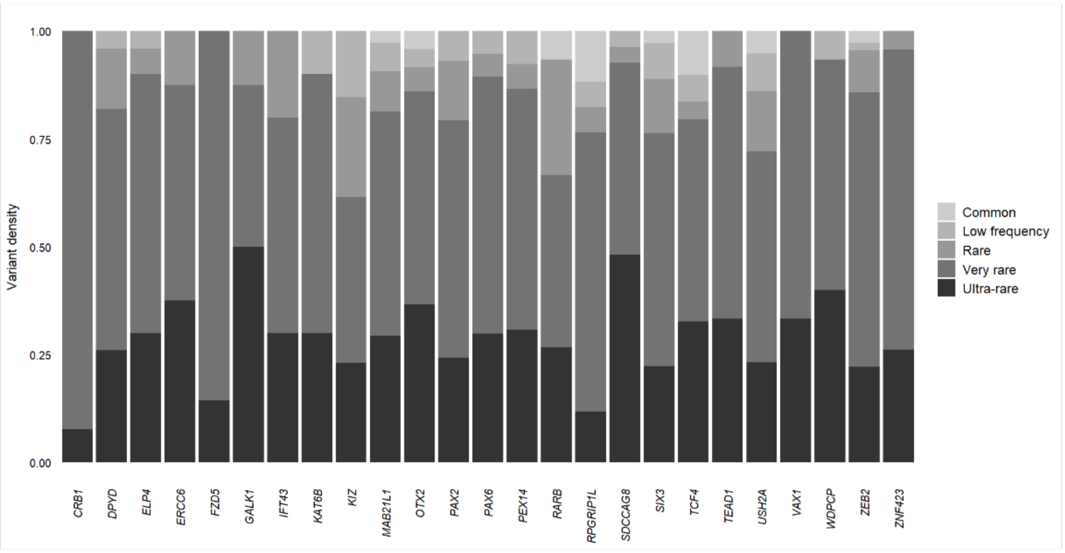

Supplementary Figure 1. Allele frequency distribution of disease-associated genes and their respective UCNEs. For a selection of 25 disease-associated genes (**a**) and their corresponding UCNEs (**b**), allele frequency distributions were generated to assess the specificity of the overlap between rare variants and UCNEs.

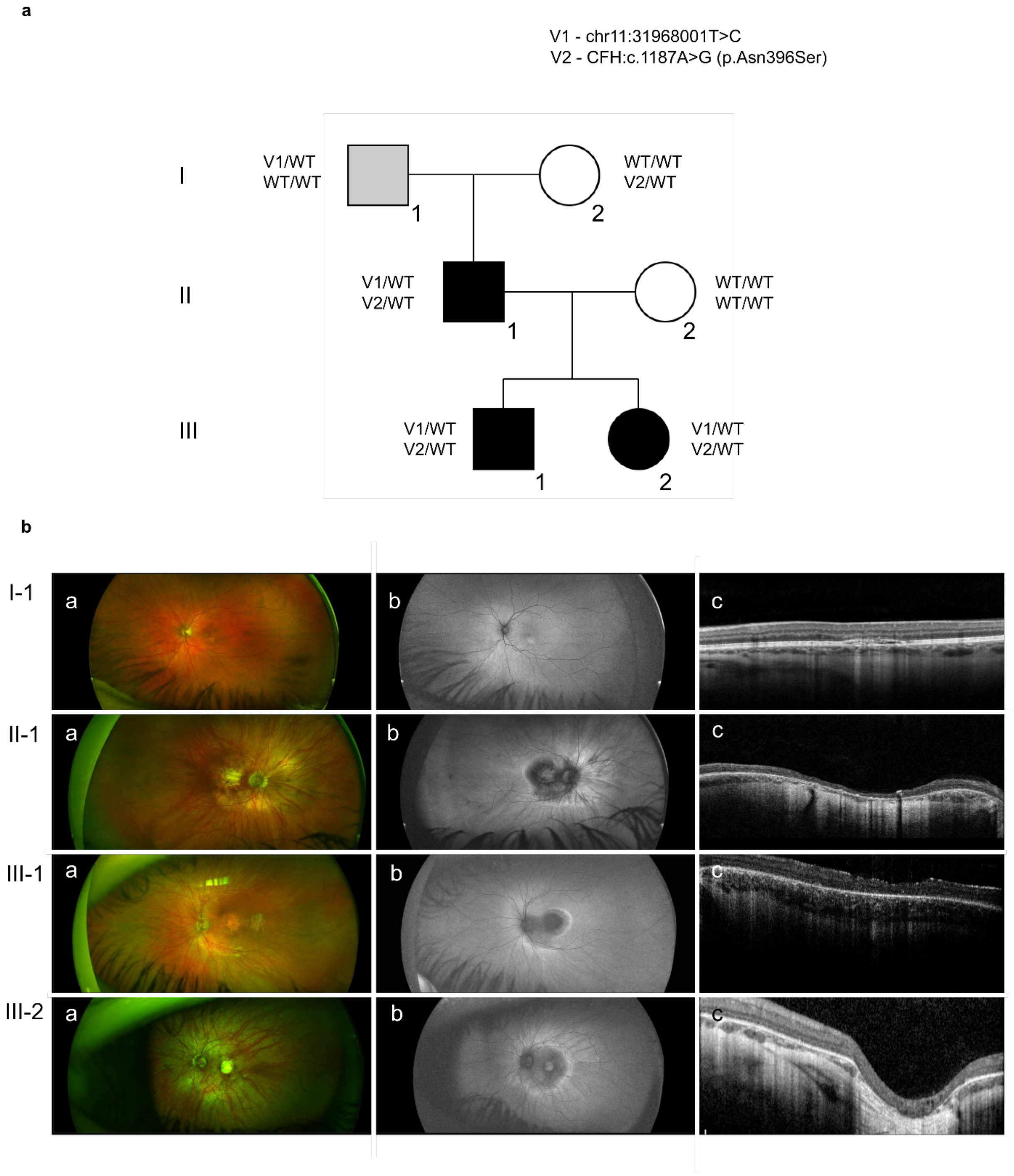

Supplementary Figure 2. *PAX6*-associated variant exclusively found in a family presenting foveal anomalies. A) Pedigree of the family, indicating segregation of the *PAX6*-associated UCNE variant (V1) and of the *CFH* variant (V2). **B)** Ophthalmological assessment (fundus examination, FAF, and OCT) revealed tessellated fundi with atrophic areas at the macula involving the fovea in II-1, III-1 and III-2, while I-1 displayed only an area of pallor inferior to the left fovea and corresponding hyper-autofluorescence (see Supplementary Data 8).

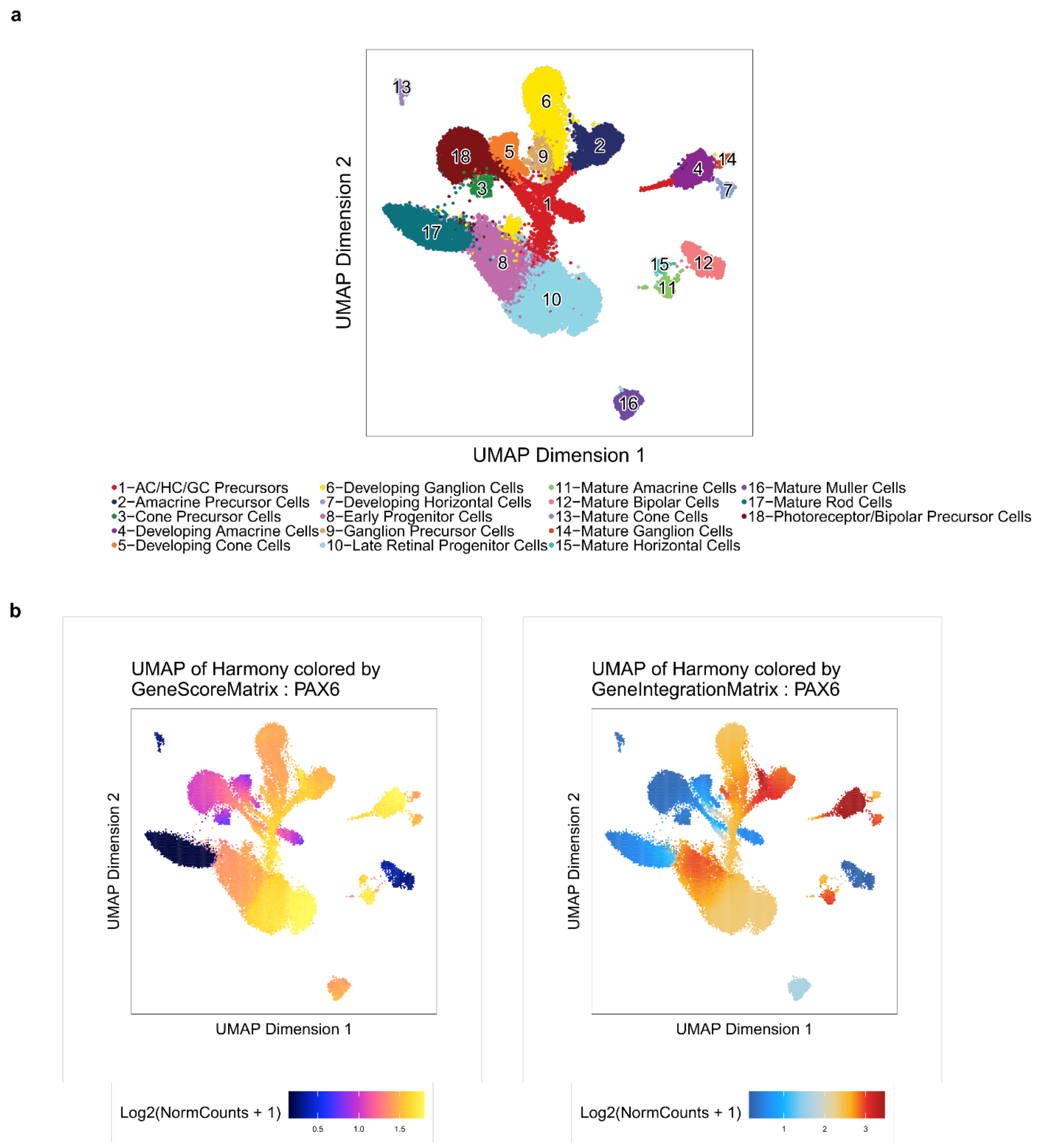

Supplementary Figure 3. Single-cell characterization of the *PAX6* gene. A) UMAP of the analyzed single cell dataset (Thomas et al., 2022).**B)** Feature plots (scATAC-seq and scRNA-seq) for the *PAX6* locus.

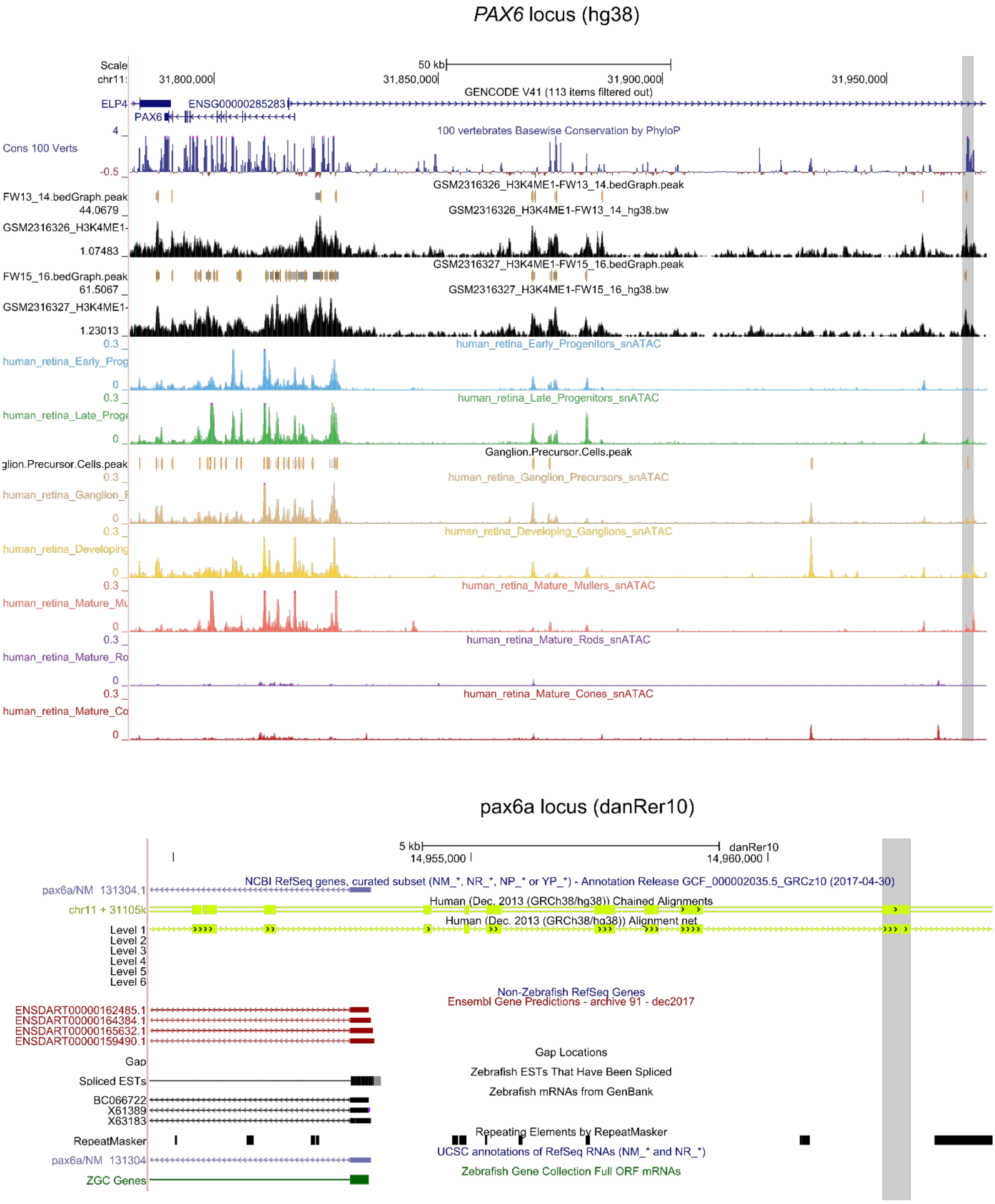

Supplementary Figure 4. Syntenic region in zebrafish of the human *PAX6* locus. Location of the characterized UCNE (*in gray*, *PAX6_Veronica*) linked to *PAX6*, found exclusively in the *pax6a* locus of zebrafish at a shorter distance (≈15kb) when compared to the human *PAX6* locus (≈150kb) with respect to the *PAX6* transcription start site.

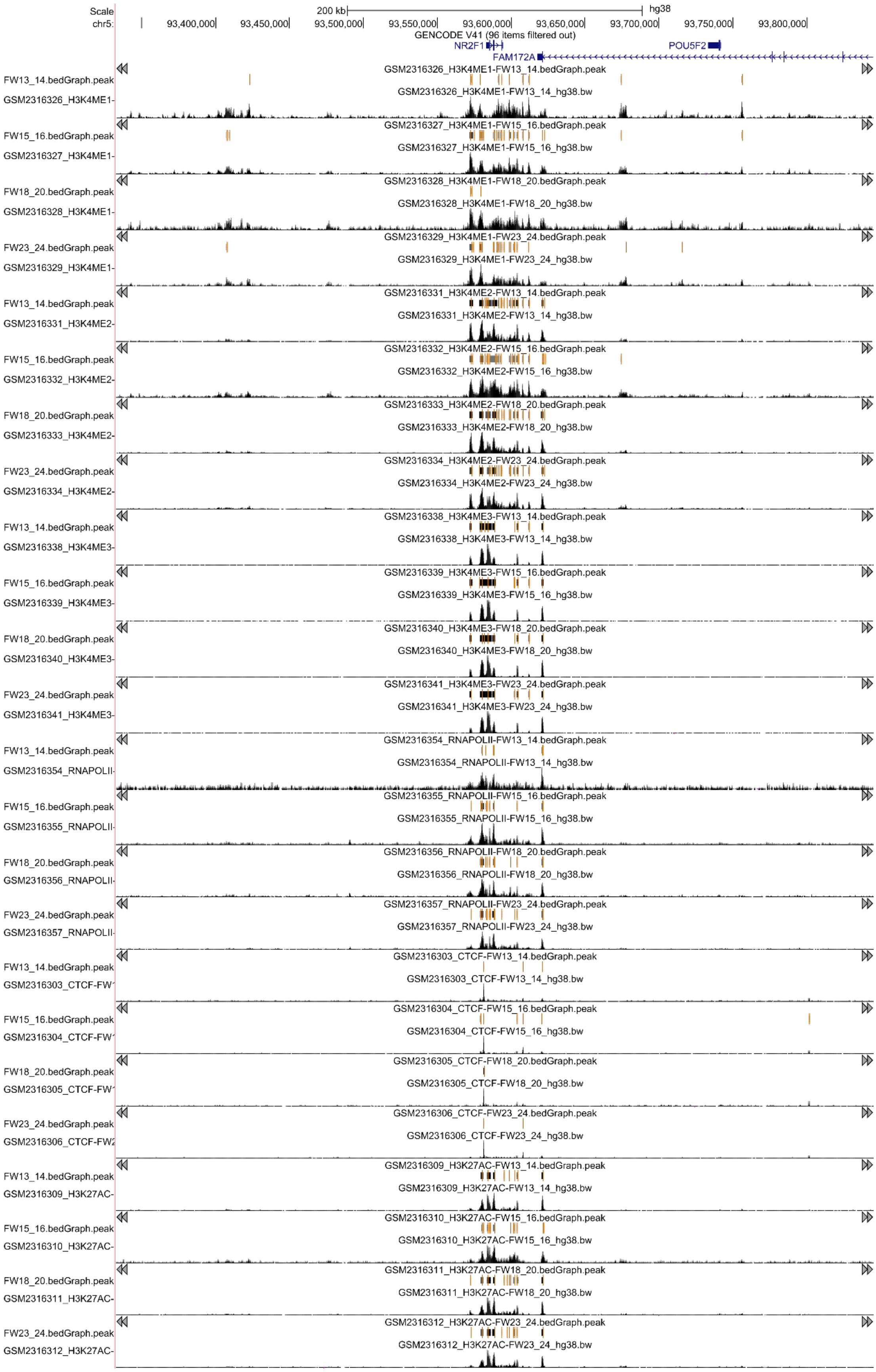

Supplementary Figure 5. Peak identification of scATAC-seq and ChIP-seq data. A) Peak identification within the *NR2F1* locus for the following marks: H3K4me1, H3K4me2, H3K4me3, CTCF, RNAPolII and H3K27ac across the different time points.

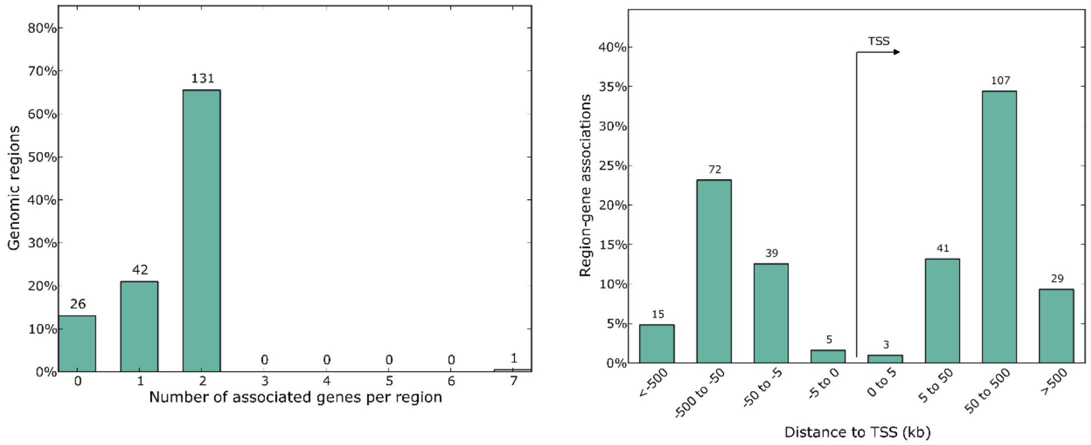

Supplementary Figure 6. Distribution of target genes associated with random genomic background. To evaluate potential confounding effects of the random genomic background, *GREAT* was used to assign their corresponding target genes. No significant differences were observed in distribution of the number of associated genes (*right*) or distances from transcription start sites (*left*) compared to those of the UCNEs (Fig. 2a-b).
