# Supplementary material for: Multi-omics analysis in human retina uncovers ultraconserved cis-regulatory elements at rare eye disease loci
Source: Nat Commun. 2024 Feb 21;15:1600. doi: 10.1038/s41467-024-45381-1 (PMC10881467; doi:10.1038/s41467-024-45381-1)
Supplement: Supplementary file 3 — Description of Additional Supplementary Files [file 41467_2024_45381_MOESM3_ESM.pdf]

Description of Additional Supplementary Files:

Supplementary Data 1: Characterization of UCNEs. UCNE ID – Original UCNE unique identifier from (Dimitrieva et al., 2013); g.coordinates (hg38) – genomic coordinate of the UCNE; Type – original classification of UCNE (Dimitrieva et al., 2013) based on the genomic location of UCNE with respect to its overlapping gene; Distance to TSS of target gene – genomic distance between UCNE and the transcription start site of its target gene (bp); Target gene – gene association extracted from GREAT analysis; Bulk RNA-seq (target gene mean expression) – expression value (TPM) obtained from the RNA-seq based on samples related to the human retinal development (Hoshino et al., 2018); Bulk RNA-seq (target gene expression rank) – expression rank obtained from the RNA-seq based on samples related to the human retinal development from (Hoshino et al., 2018); Bulk RNA-seq (target gene maximum expression) – maximum values of gene expression (TPM) obtained from the RNA-seq based on samples related to the human retinal development (Hoshino et al., 2018); Bulk RNA-seq (stage of maximum target gene expression) – stage where the maximum levels of gene expression where observed, obtained from the RNA-seq based on samples related to the human retinal development (Hoshino et al., 2018); scRNA-seq target gene expression – retinal cell cluster where the expression of the target gene is expected, obtained from the scRNA-seq based on samples related to human retinas (Thomas et al., 2022); DNase-seq (stage) – stages where the open chromatin context was identified, obtained from the DNase-seq experiments related to the retinal development (ENCODE); scATAC-seq – cell clusters where the peak identification was retrieved (Thomas et al., 2022); Retinal TAD Support – qualitative estimation of the association UCNE-target gene; ChIP-seq - epigenomic marks, CTCFs and PolII peaks observed within the genomic context of the characterized UCNE (Aldiri et al., 2017 , Cherry et al., 2020); VISTA (Element ID) – assessment of the inclusion of the UCNE element within the VISTA enhancer browser and its unique ID; VISTA (Assay result) – reporter assay result from the VISTA enhancer browser; VISTA (Expression pattern) – includes the tissues where the expression pattern for the reporter assay was observed.

Supplementary Data 2: Characterization of UCNEs based on epigenomic marks.

Supplementary Data 3: Full gene set ontology enrichment results from EnrichR.

Supplementary Data 4: Comparison between target genes assigned by GREAT and peak-to-gene linkage method.

Supplementary Data 5: Disease association of UCNE target genes. #OMIM disease name; confidence category; allelic requirement; mutation consequence; phenotypes; organ specificity list; PMIDs. Additional genomic and functional annotations are the same as in Table S1.

Supplementary Data 6: Overview of the eye disease sub-cohort of 100,000 Genomes Project (Genomics England). Normalized Disease Group, subgroup, and specific disease; Participant Count.

Supplementary Data 7: Variants retrieved within the UCNEs that are linked to an eye or retinal disease phenotype. It includes the gene, the UCNE ID and its coordinates, and the retrieved variant (hg38).

Supplementary Data 8: Phenotypic description of the studied family segregating autosomal dominant foveal abnormalities. Participant information (ID, origin and sex), molecular findings (carriers of the V1 (chr11:31968001T>C) and/or V2 (CFH, c.1187A>G (p.Asn396Ser)), diagnosis and clinical findings.

Supplementary Data 9: Analysis of the TFBS motif disruption potentially exerted by the chr11:31968001T>C variant (qBiC-PRED) and retrieved variants within genes associated with macular developmental defects and foveal hypoplasia (IRX1, PRMD13, SLC38A8, GPR143, FRMD7 and AHR). Output from qBiC-PRED includes the predicted TF binding changes, associated name in protein binding microarray experiments (pbmname), the normalized changes (z-scores), the significance of the changes according to each model (p.value), and the predicted changes in binding status (e.g. bound > unbound).

Supplementary Data 10: Overview of the transgenic enhancer assays in zebrafish for the PAX6-associated UCNE (PAX6_Veronica).

Supplementary Data 11: Overview of the datasets used in this study.

Supplementary Data 12: Set of primers used in this study for cloning and segregation.
